# Investigating the role of oviductal mucosa–endometrial co-culture in modulating factors relevant to embryo implantation

**DOI:** 10.1515/med-2024-1077

**Published:** 2024-12-04

**Authors:** Chengrong Wu, Hualei Cai, Qian Pu, Lei Yu, Ashutosh Goswami, Zhongyuan Mo

**Affiliations:** Assisted Reproductive Center, Guiyang Women’s and Children’s Hospital (Guiyang Children’s Hospital), Guiyang, 550000, China; Department of Obstetrics and Gynecology, The Affiliated Hospital of Guizhou Medical University No. 28 of Guiyi Street, Yunyan District, Guiyang, Guizhou, 550000, China; Department of Obstetrics and Gynecology, Guiyang Women’s and Children’s Hospital (Guiyang Children’s Hospital), Guiyang, 550000, China; Department of Orthopaedics, The Affiliated Hospital of Guizhou Medical University, Guiyang, 550000, China; Department of Paediatrics, Guizhou Provincial General Hospital of the Armed Police, Guiyang, 550000, China

**Keywords:** uterine adhesion, tubal mucosa, endometrium, co-culture, endometrial tolerance

## Abstract

**Background:**

Intrauterine adhesions (IUAs) are a significant clinical challenge, affecting reproductive health and leading to infertility or recurrent pregnancy loss. Understanding the molecular mechanisms underlying IUA prevention is crucial for developing effective treatment strategies.

**Objective:**

To investigate the interaction between oviductal mucosal cells and endometrial cells and their effects on the expression of key molecules involved in embryo implantation, specifically leukemia inhibitory factor (LIF), avβ3, estrogen receptor (ER), and progesterone receptor (PR).

**Methods:**

Tubal mucosa and endometrium specimens were collected from 22 patients undergoing surgical interventions. Cells were cultured alone and co-cultured at ratios of 1:1, 1:0.5, and 1:0.1. LIF, avβ3, ER, and PR expression levels were measured using real-time fluorescence quantitative polymerase chain reaction and enzyme-linked immunosorbent assay.

**Results:**

Our results demonstrated that LIF expression was significantly augmented in co-culture conditions, particularly in the 1:1 ratio, compared to oviductal mucosa monoculture (*P* < 0.05). Although LIF expression was also elevated in 1:0.5 and 1:0.1 co-culture ratios, these increases were not statistically significant (*P* > 0.05). For avβ3, increased expression was observed in the 1:1 co-culture group (*P* < 0.05), but no significant differences were detected in 1:0.5 and 1:0.1 co-culture groups. ER expression showed a downward trend in co-culture, but without statistical significance (*P* > 0.05), and PR expression remained stable across all groups.

**Conclusion:**

Co-culture modulates key molecules involved in embryo implantation, particularly LIF and avβ3. These findings highlight the potential roles of LIF and avβ3 in IUA prevention strategies and provide important insights for future clinical interventions. Tubal mucosal cells can not only grow in the endometrial cell microenvironment, but also the tolerance of tubal mucosal cells can be improved when they are co-cultured.

## 1 Introduction

Infertility remains a significant global public health issue, affecting approximately 10–15% of couples worldwide, with a rising trend in recent years [1]. Over the past years, China has witnessed an accelerated decline in its population, prompting the government to sequentially introduce the two-child and then the three-child policies to boost demographic growth [2]. Despite these efforts, the country’s natural growth rate has fallen short of expectations, with a persistent downward trend in the birth rate. Consequently, addressing the fertility rate decline has become a pressing concern in China [3]. Notably, infertility is now recognized as one of the world’s top three public health issues, affecting 10–15% of couples globally, with a rising trend in recent times [4]. According to a recent study by the World Health Organization, the global prevalence of infertility has increased by approximately 2% per year over the past decade [5]. Alongside this, the infertility rate among the Chinese population is higher than anticipated, contributing to the declining birth rate annually. In China, the prevalence of infertility is estimated to be around 12%, with a significant proportion attributed to uterine factors [6].

Among the various causes of infertility, intrauterine adhesions (IUAs) are a leading cause [7]. IUAs arise from infection or trauma, resulting from damage to the endometrium’s basal layer due to diverse factors such as repeated abortions and infections [8]. This leads to a scarcity of cells for endometrial regeneration, fibrosis, unregulated extracellular matrix deposition, and substitution of the interstitium with fibrous connective tissue, along with glands being replaced by inactive cuboidal columnar epithelium [9]. Symptoms often include reduced menstrual flow, amenorrhea, abdominal pain, or infertility. Hysteroscopic transcervical resection of adhesions is the principal treatment for IUA, followed by hormonal therapy, vasodilatory agents, and non-degradable stent insertion as common postoperative adjunct therapies [10]. However, these interventions have limited efficacy in preventing adhesion recurrence and preserving fertility. Studies have shown that the recurrence rate of IUAs after surgical intervention ranges from 21 to 62.5%, with a significant impact on reproductive outcomes [11].

These rare instances of surviving ectopic pregnancies and even the delivery of live offspring [9,10] suggest that alternative sites, albeit unconventional, can support implantation. This implies that while the uterine endometrium is uniquely suited for timed embryo implantation, allowing only a specific window for this process, other tissues may possess certain capabilities for implantation under extraordinary circumstances. Given the challenges faced by patients with moderate to severe IUA, seeking expanded therapeutic strategies to restore fertility is crucial. Our research team initially posited the concept of using fallopian tube mucosa as an alternative to endometrial tissue. Our previous studies have shown that tubal mucosa cells can coexist harmoniously with endometrial cells, and that these coexisting tubal mucosa cells exhibit cell passage potential [11]. This innovative approach aims to broaden the treatment landscape for IUA patients and potentially facilitate successful pregnancy outcomes. Indeed, a successful pregnancy not only requires a repaired uterine environment but also a receptive endometrium, the key factor in embryo acceptance. Endometrial receptivity acts as a biological marker of embryo quality, rejecting low-quality embryos. While embryo quality contributes to about one-third of implantation failures, the majority, two-thirds, are due to inadequate endometrial tolerance [12–15]. The endometrium serves as the final barrier in assisted reproductive technologies (ART), and poor tolerance is the primary reason for ART-related implantation failures [16].

Given the clinical significance of IUAs and the limitations of current treatments, there is a critical need to explore new and effective therapeutic targets for the treatment of IUA. This study aims to explore the interaction between oviductal mucosal cells and endometrial cells using real-time fluorescence quantitative polymerase chain reaction (PCR) and enzyme-linked immunosorbent assay (ELISA) to better understand the change of key molecules such as leukemia inhibitory factor (LIF) and avβ3 in tubal mucosal cells during co-culture.

## 2 Materials and methods

### 2.1 Sample section

The tubal mucosa and endometrium specimens used in this study were collected from 22 patients who underwent endometrial scraping and removal of fallopian tubes and uterus due to infertility, uterine fibroids, adenomyosis, CIN III, and other conditions requiring surgical intervention. The collections took place at the Department of Gynecology, Affiliated Hospital of Guizhou Medical University, from November 2019 to December 2020.

#### 2.1.1 Inclusion criteria

Inclusion criteria include: (1) uterus and fallopian tubes needed to be removed due to benign gynecological diseases, (2) women of reproductive age, (3) no steroid hormone was taken 3 months before surgery, (4) postoperative pathological examination confirmed no malignant pathological changes in fallopian tubes and endometrium, (5) surgery was performed at the early and middle stages of hyperplasia, and (6) patients gave informed consent and signed the informed consent form.

#### 2.1.2 Exclusion criteria

Exclusion criteria include: (1) gynecological malignancy; (2) postoperative pathological examination of the fallopian tubes or endometrium with malignant or precancerous lesions; (3) hormonal drugs within 3 months after surgery; (4) postmenopausal women; (5) patients with combined renal disease, hyper- or hypothyroidism, and immune diseases; and (6) patients who did not agree to the use of specimens for this experiment.

### 2.2 Experimental trials and grouping

The cells were co-cultured in the following groups: (A) fallopian tube mucosal cells (control group), (B) endometrial cells (upper chamber) + oviductal mucosal cells (lower chamber) with a 1:1 ratio of cells in the upper chamber: lower chamber (experimental group 1), (C) endometrial cells (upper chamber) + oviductal mucosal cells (lower chamber) with a 0.5:1 ratio of cells in the upper chamber: lower chamber (experimental group 2), and (D) endometrial cells (upper chamber) + oviductal mucosal cells (lower chamber) with a 0.1:1 ratio of cells in the upper chamber: lower chamber (experimental group 3).

### 2.3 *In vitro* analysis and cultivations

After removing the uterus and fallopian tubes from the body, the endometrium and separated fallopian tube mucosa were immediately collected in sterile specimen bottles containing saline, stored at 4°C, and sent to the laboratory within 1 h. The specimens were removed and placed in a 3.5 cm diameter petri dish, washed thrice with phosphate-buffered salt (PBS) solution to remove the surface red blood cells, tissues were cut into small pieces less than 1 mm, transferred to 15 mL centrifuge tubes with pipettes, washed once with PBS solution, centrifuged at 1,200 rpm for 5 min, and the supernatant was discarded; type I collagenase (C0103, Sigma) was added separately. About 0.1 mg/mL was added for digestion and placed in a constant temperature water bath at 37°C, during which the tubes were shaken every 10 min, and the digestion was terminated by adding DMEM/F12 after 90 min.

The endometrial suspension and oviductal mucosa suspension were centrifuged at 1,200 rpm for 5 min, and the supernatant was removed to obtain the precipitates. Then 10% fetal bovine serum (extra fetal bovine serum (South America), 04-001-1ACS, BI) complete culture solution was added to both precipitates, blown and mixed, and inoculated into cell culture flasks, then the endometrial and oviductal mucosa cell culture flasks were incubated at 37°C CO_2_ thermostat for 24 h to observe the cell apposition and growth, and if the cells were well opposed to the bottom and growing, the fluid was changed every 48 h and the cell morphology was observed by inverted microscope.

When the cells grew to 80–90% of the bottom of the culture flask, the original medium was discarded and washed twice with PBS, 0.25% trypsin was added to just cover the bottom of the flask, and the flask was placed in 37°C 5% CO_2_ incubator for 1–2 min to digest. Added 10% fetal bovine serum complete medium to the cell precipitate, mixed well by blowing, inoculated with 10^5^ cells/mL cell suspension in culture flasks for cell passaging, and replenished with 10% fetal bovine serum complete medium to 6 mL in culture flasks, and placed them in a 37°C 5% CO_2_ incubator.

### 2.4 Cell co-culture analysis

The fifth-generation oviductal mucosa cells in the logarithmic growth phase with good growth status were inserted into the lower chamber of the six-well plate with 1.8 × 10^5^ cells/well, while the fifth-generation endometrial cells in logarithmic growth phase with good growth status were inserted into the upper chamber of six-well plate with 1.8 × 10^5^, 9 × 10^4^, 1.8 × 10^4^ cells/well (the membrane pore size of transwell chamber is 0.4 μm, so the cells cannot pass through). After the cells were attached to the wall, the endometrial cells were first cultured in cell culture medium with 2% fetal bovine serum, and co-cultured with oviductal mucosal epithelial cells; after 48 h of culture, the medium was changed to that for inducing cell metaphase (inducing cell metaphase medium). Ingredients: 2% fetal bovine serum cell culture medium, 1 μm progesterone (V900699, Sigma), 10 nM 17-β-estradiol (E110145, Aladdin), 0.5 mM 8-bromoadenosine 3′,5′-cyclic monophosphate (B5386, Sigma) were continued to be cultured for 96 h; after metaphase cell growth the changes of cells were observed under inverted microscope.

### 2.5 Gene expression analysis using PCR

The real-time fluorescence quantitative PCR method was used to detect the expression levels of mRNA of avβ3 and LIF in oviductal mucosal cells in the lower chamber of each group: oviductal mucosal cells in each group after co-culture and metaphase treatment as previously grouped, total RNA was extracted with Trizol reagent instructions for concentration determination, quantification, reverse transcription, and amplification. The primers were synthesized by Beijing Prime Tech Biotechnology Co. β-actin was used as the internal reference, and the relative expression was calculated according to 2^−△△Ct^. The primer sequences are detailed in Table 1.

**Table 1:** Primer sequences with their product size

Gene		Sequence	Product (bp)
Homo b-actin	Forward	CCCTGGAGAAGAGCTACGAG	180
	Reverse	CGTACAGGTCTTTGCGGATG	
Homo avβ3	Forward	TGGGGCTGATGACTGAGAAG	206
	Reverse	ACGCACTTCCAGCTCTACTT	
Homo LIF	Forward	CCAACAACCTGGACAAGCTATG	118
	Reverse	GTGCCAAGGTACACGACTATGC	

### 2.6 Western blot analysis

The oviductal mucosal cells grouped as before were taken, extracted the total protein after sufficient lysis, determined the protein concentration by BCA method, then performed SDS-PAGE electrophoresis, transferred the membrane, close the transferred membrane with 5% skimmed milk for 1 h, diluted the primary antibody (estrogen receptor-alpha antibody; Affinity, Af6058, 1:1,000 and progesterone receptor; Affinity, Af6106, 1:1,000 dilution), put overnight at 4°C, washed with TBST, then diluted the secondary antibody 10,000 times with TBST, incubated for 1 h at room temperature, washed again with TBST, dropwise added equal volume of mixed ECL developer, and put into the gel imager for exposure imaging. Finally, the grayscale values were analyzed using Image J 1.8.0 software, with β-actin as the internal reference.

### 2.7 ELISA

The supernatant of each group of cell culture was collected and centrifuged at 3,000 rpm for 10 min, and the supernatant was taken to detect the secretion of human LIF. The procedure was performed according to the instructions of the reagent vendor (Elabscience^®^). The optical density of each group was measured at 450 nm using an enzyme standardization instrument.

### 2.8 Immunohistochemistry (IHC) analysis

Cell crawling was performed as in our previously published article. After crawling with 4% paraformaldehyde fixed for 15 min and 0.5% Triton X-100 (PBS preparation) permeabilized at room temperature for 20 min, antigen repair and closure were performed, and 100 μL of diluted primary antibody (estrogen; Affinity, AF6058, 1:100) (αv + β3; Bioss,bs-1310R, 1:100) was added dropwise to each section), put to rest overnight at 4°C, rinsed with PBS; then 100 μL of biotin-labeled secondary antibody working solution (1:150 dilution) was added dropwise, incubated at 37°C for 30 min, rinsed with PBS. Added DAB chromogenic solution dropwise, developed color at room temperature, re-stained, returning blue, dehydrated transparently, dried with air and sealed the film, placed under a microscope, and observed and photographed the results with Imagepro-Plus to detect the average optical density (AOD) value of the pictures.

### 2.9 Statistical analysis

All statistical analyses were performed using GraphPad (version 7.0; GraphPad Software, Inc.) and SPSS (22.0; IBM, Corp.), and measures conforming to a normal distribution were expressed as mean ± standard deviation, and comparisons of measures between multiple groups conforming to a normal distribution with chi-square were performed using one-way ANOVA analysis, and two-way comparisons between groups were performed using the Dunnett test. Dunnett’s test was used for comparison between groups, and the Kruskal–Wallis H method was used for non-conformity.


**Informed consent:** Patients gave informed consent and signed the informed consent form.
**Ethics approval:** This study was approved by the Ethics Committee of the Affiliated Hospital of Guizhou Medical University (No. 2020-(264-01)).

## 3 Results

Endometrial and fallopian tube mucosal cell identification and metaphase manifestations have been shown in detail in our previous published articles [11] and results were presented in detail.

### 3.1 Comparative expression of LIF and avβ3 in co-culture and monoculture of oviductal mucosa and endometrial cells

Endometrial tolerance is influenced by expression of LIF in the key molecule for embryo implantation. Changes in avβ3 expression may affect cell adhesion and embryo implantation. To demonstrate the effect of intercellular interaction on LIF and avβ3 expression when tubal mucosal cells were co-cultured with endometrial cells.

**Figure 1:**
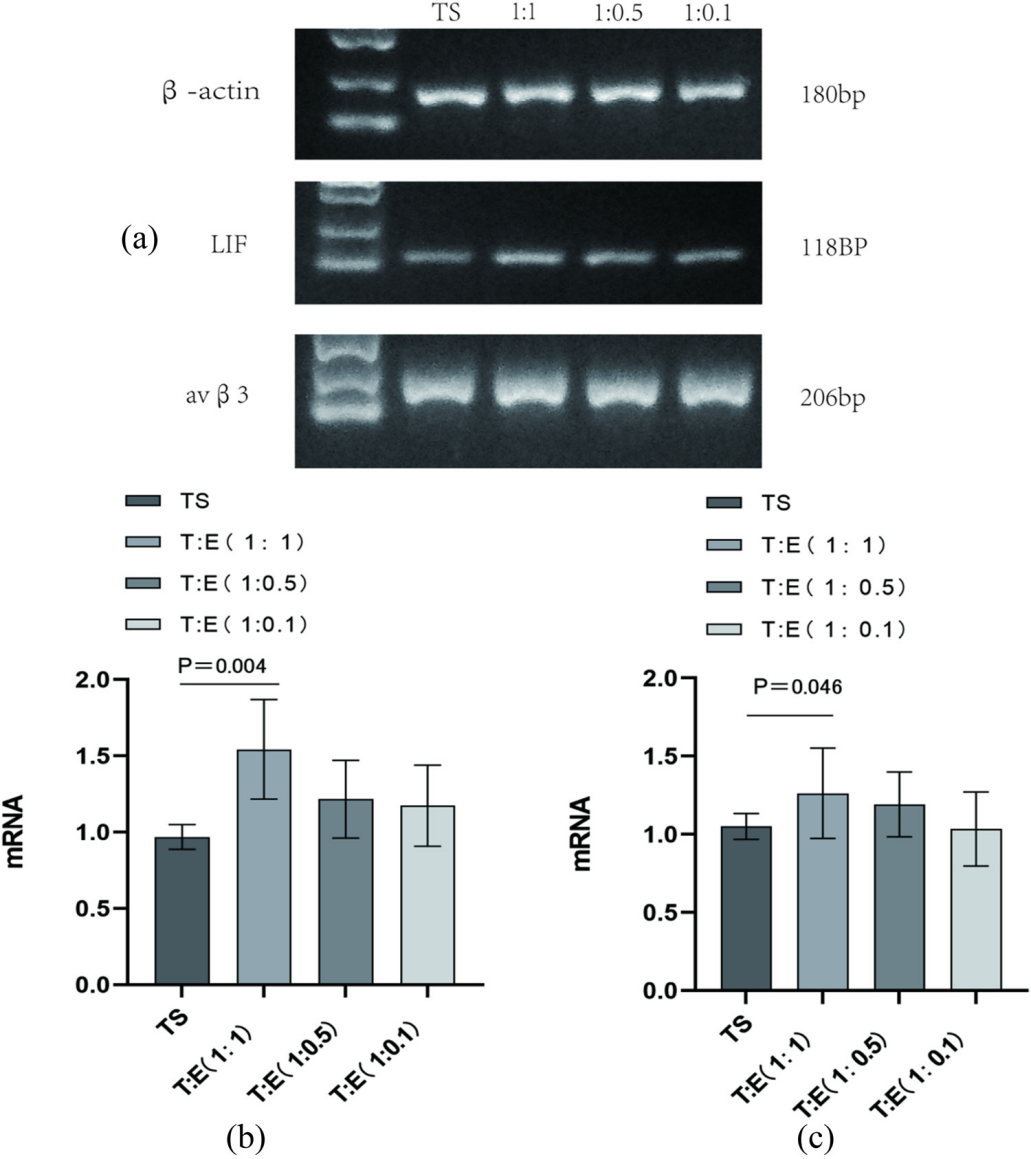
(a) and (b) Compared with the tubal mucosal cell alone culture group (control group), avβ3 expression was higher in the tubal mucosal cell and endometrial cell (1:1) co-culture group (experimental group 1), *P* < 0.05, with statistical differences. (a) and (c) (1:0.5, 1:0.1) Co-culture (experimental groups 2 and 3) avβ3 expression *P* > 0.05, without statistical difference. TE: tubal mucosal cell and endometrial cell; TS: tubular mucosal cells single.

Our PCR results showed that LIF expression was notably higher in the group co-culturing oviductal mucosa with endometrial cells (group 1) compared to the solo oviductal mucosa group (control), with a strong statistical difference (*P* < 0.01). However, LIF levels in the 1:0.5 and 1:0.1 co-culture groups (groups 2 and 3) were also upregulated, but not significantly as compared to the control (*P* > 0.05) (Figure 1a and b). For avβ3, its expression was increased in the 1:1 combined culture of tubal mucosa and endometrial cells (group 1) versus the tubal mucosa-only group (control), with a statistically significant difference (*P* < 0.05) (Figure 1a and c). There was no significant difference in avβ3 expression between the 1:0.5 and 1:0.1 co-culture groups and the control (*P* > 0.05).

### 3.2 Quantitative analysis of LIF expression via ELISA in oviductal mucosa–endometrial cell co-culture systems

**Figure 2:**
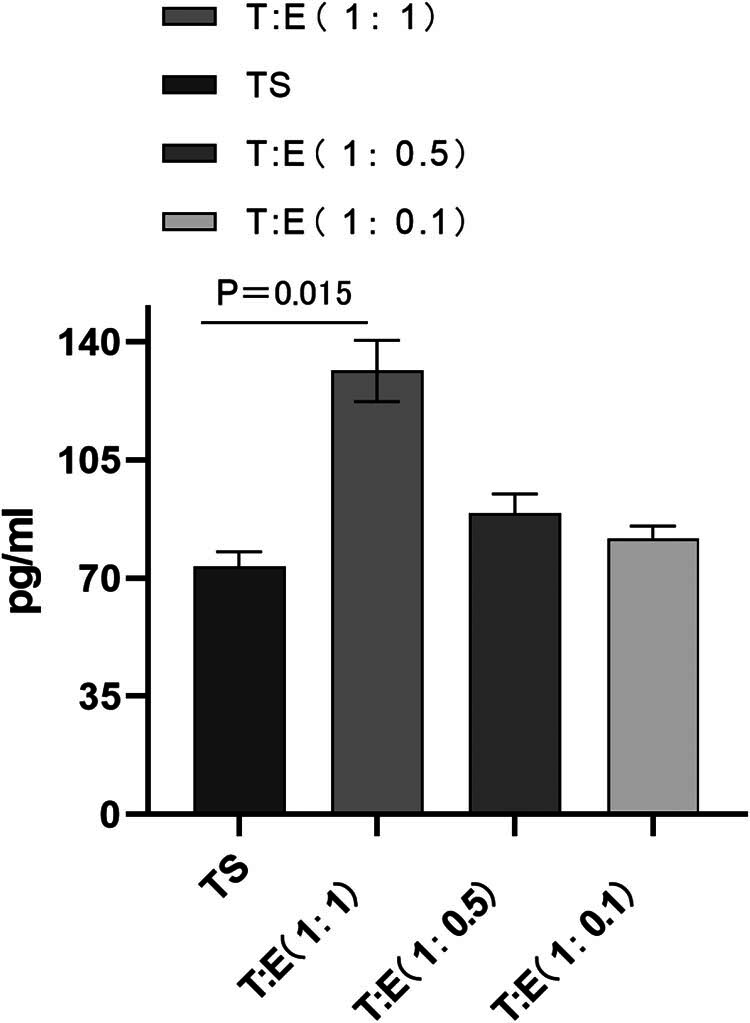
ELISA detection showed that the expression of LIF in the co-culture group of oviduct mucosal cells and endometrial cells (experimental group 1) was also significantly higher than that in the simple oviduct mucosal cells group (control group), *P* < 0.05, the expression of LIF in the co-culture group (experimental groups 2 and 3) (1:0.5 and 1: 0.1) was also higher than that in the control group, but *P* > 0.05, the difference was not statistically significant. TE: tubal mucosal cell and endometrial cell; TS: tubular mucosal cells single.

To substantiate the LIF expression results, we conducted an ELISA, revealing that LIF levels were indeed significantly augmented in the co-culture of tubal mucosa and endometrial cells (experimental group 1) relative to the solitary tubal mucosa group (control), with a statistically significant difference (*P* < 0.05). Moreover, in the 1:0.5 and 1:0.1 co-culture conditions (groups 2 and 3), LIF expression was also higher compared to the control, though the differences were not statistically significant (*P* > 0.05) (Figure 2).

### 3.3 Estrogen receptor (ER) downregulation and progesterone receptor (PR) stability in oviductal mucosa–endometrial cell co-culture models

**Figure 3:**
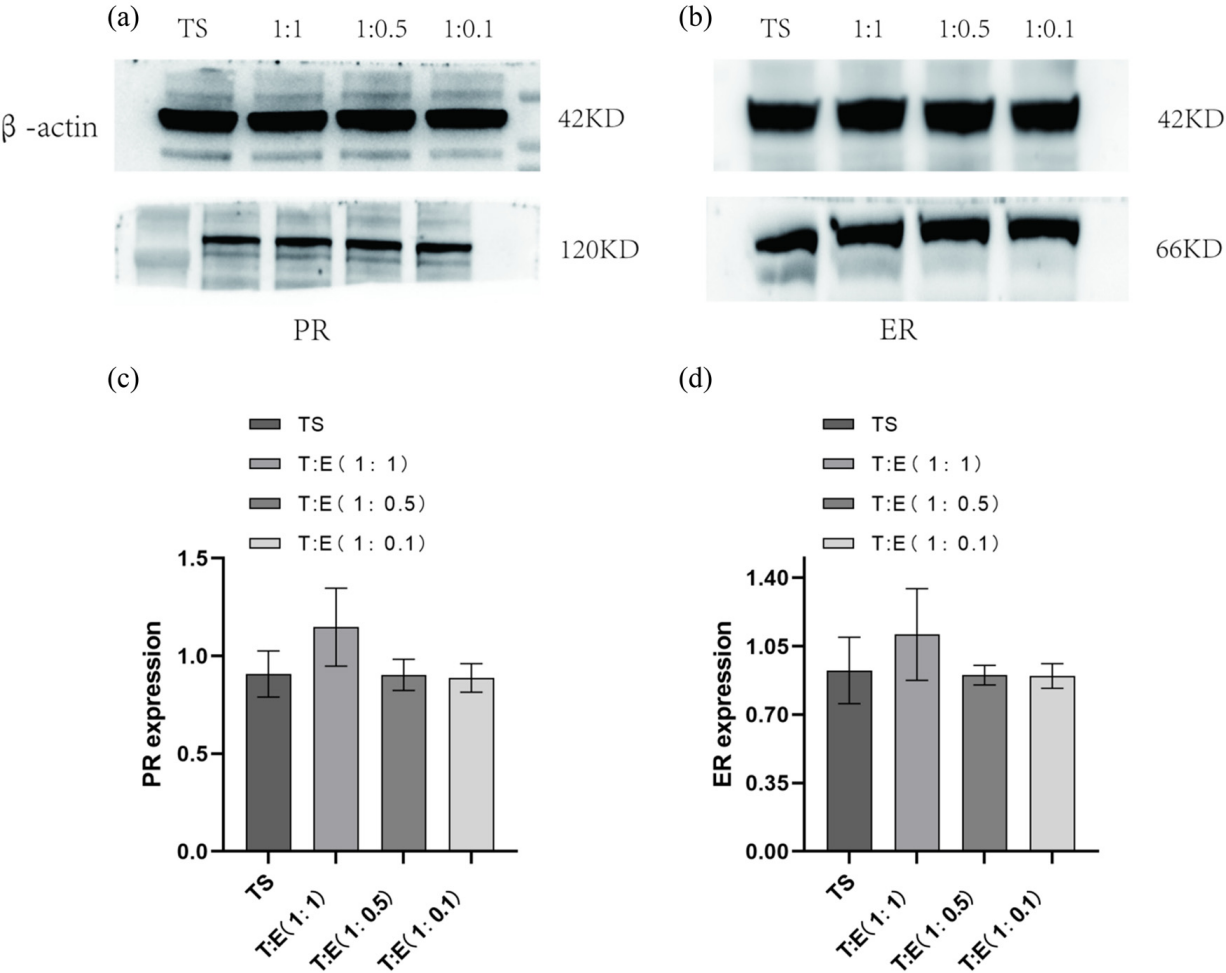
Expression of ER was lower in both tubal mucosal cells alone than in co-culture with endometrial cells (1:1, 1:0.5, and 1:0.1 ratios; experimental groups 1, 2, and 3), but the *P* value was >0.05, no statistical difference (b) and (d). Expression of PR was close to that in tubal mucosal cells alone and co-culture with endometrial cells (1:1, 1:0.5, and 1:0.1 ratio) were close to each other, and there was no significant difference between the groups, *P* > 0.05, no statistical difference (a) and (c). Intrauterine adhesions: IUAs; PCR: polymerase chain reaction; ELISA: enzyme-linked immunosorbent assay; LIF: leukemia inhibitory factor; ER: estrogen receptor; TE: tubal mucosal cell and endometrial cell; TS: tubular mucosal cells single.

Observations of ER expression indicated a discernible downregulation in tubal mucosa cells cultivated independently, as opposed to their co-culture with endometrial cells at ratios of 1:1, 1:0.5, and 1:0.1 (designated as experimental groups 1, 2, and 3). However, these differences did not attain statistical significance (*P* > 0.05) (Figure 3b and d). Conversely, PR expression demonstrated a remarkable similarity between solitary tubal mucosa cells and those co-cultured with endometrial cells across the same ratios, with no statistically appreciable disparities noted between the groups (*P* > 0.05) (Figure 3a and c).

### 3.4 Immunohistochemical validation of avβ3 expression and ER stability in oviductal mucosa–endometrial co-culture

**Figure 4:**
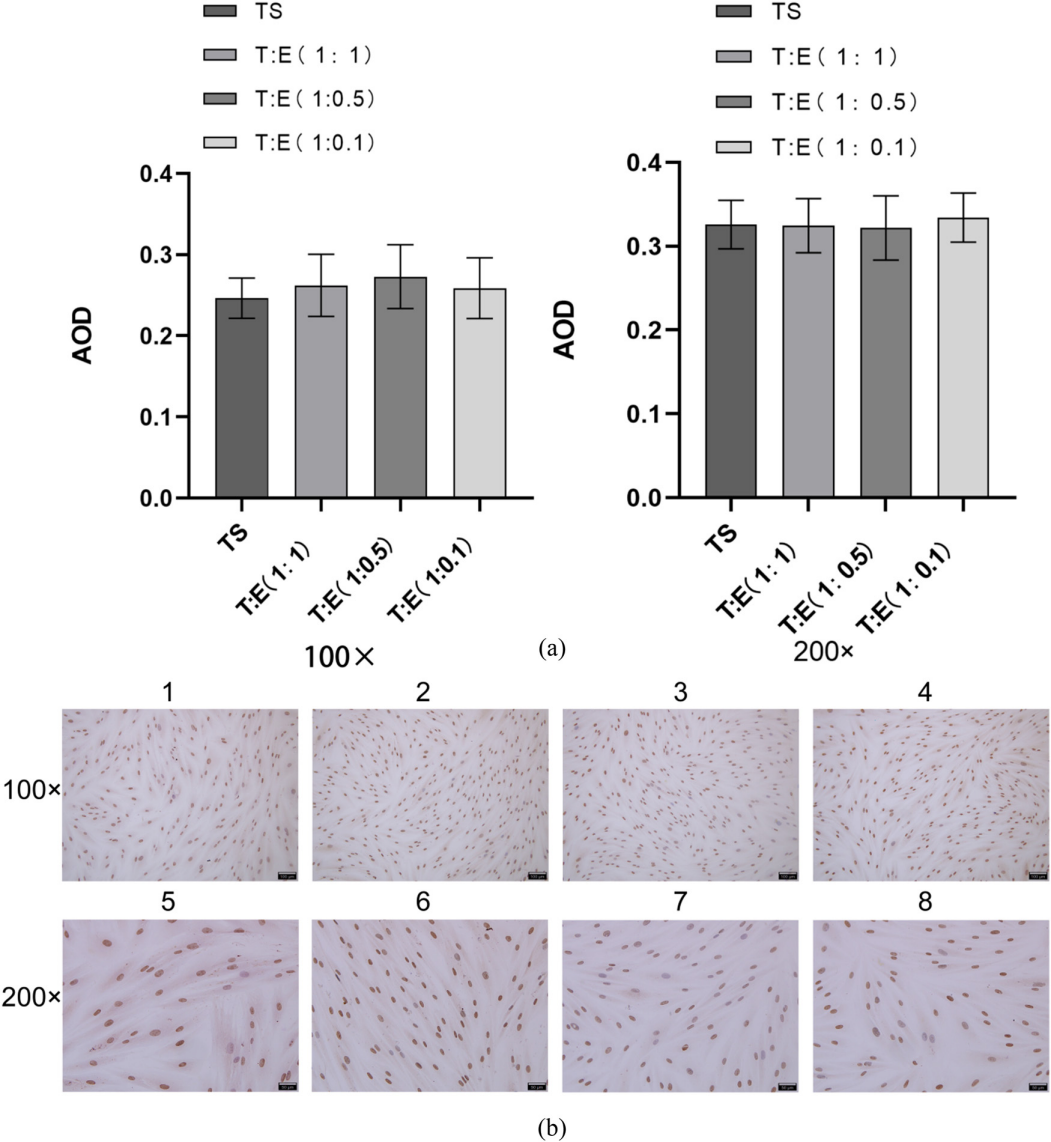
Immunohistochemical assay to detect ER expression in each group of cells. (a) Quantitative comparison of mean optical density values (AOD) of ER expression in each group of cells. (b) Immunohistochemical results of ER in each group of cells, blue is the nucleus and tan or brown is the target protein expression. Intrauterine adhesions: IUAs; PCR: polymerase chain reaction; ELISA: enzyme-linked immunosorbent assay; LIF: leukemia inhibitory factor; ER: estrogen receptor; TE: tubal mucosal cell and endometrial cell; TS: tubular mucosal cells single.

**Figure 5:**
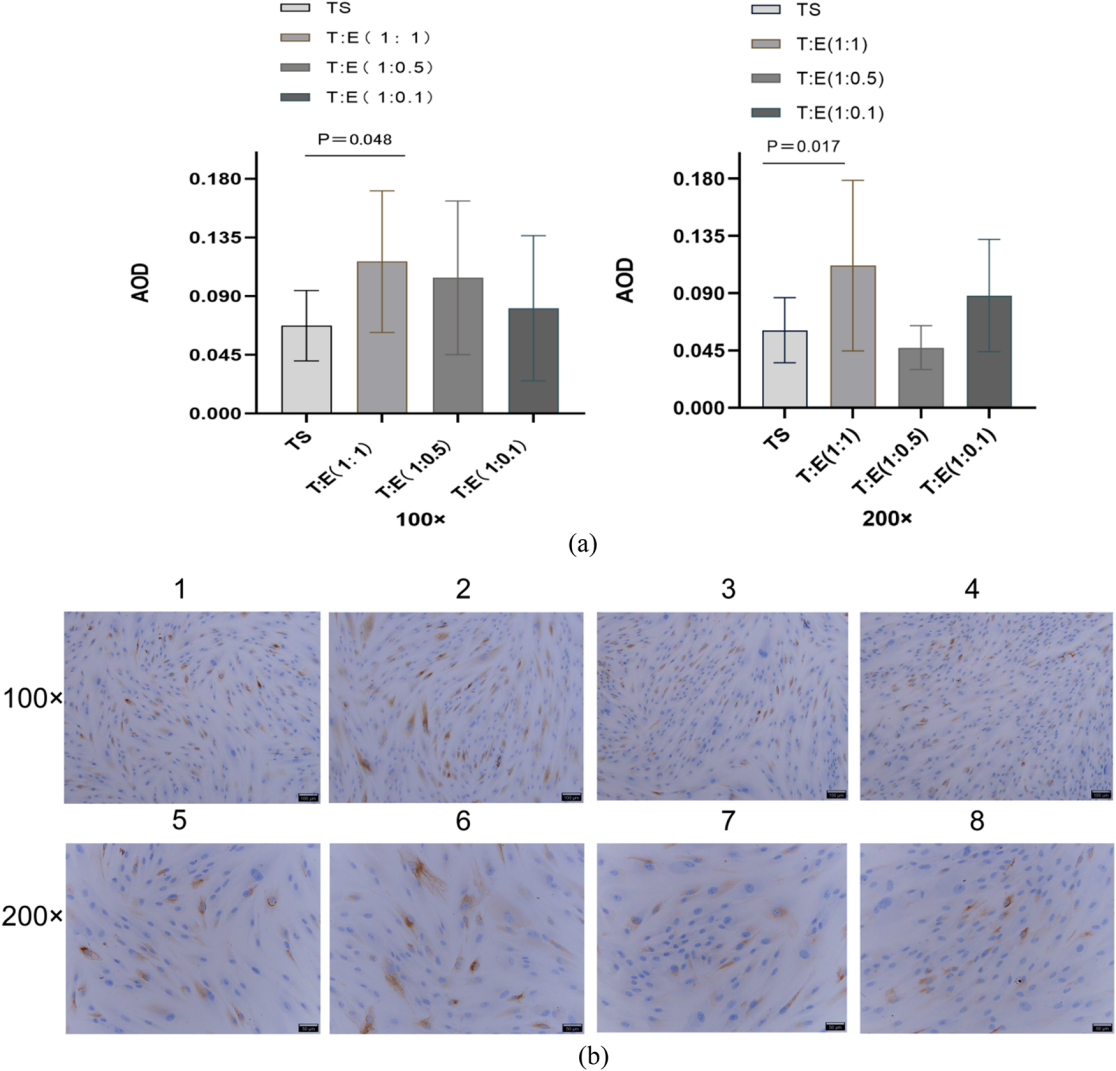
Immunohistochemical assay to detect the expression of avβ3 in each group of cells. (a) Quantitative comparison of mean optical density values (AOD) of avβ3 expression in each group of cells. (b) Immunohistochemical results of avβ3 in each group of cells, blue is the nucleus, and tan or brown is the target protein expression. Intrauterine adhesions: IUAs; PCR: polymerase chain reaction; ELISA: enzyme-linked immunosorbent assay; LIF: leukemia inhibitory factor; ER: estrogen receptor; TE: tubal mucosal cell and endometrial cell; TS: tubular mucosal cells single.

To further validate the expression patterns of avβ3 and ER, we conducted post-climb immunohistochemical analyses on each cellular group. Our findings aligned with the PCR outcomes, demonstrating that avβ3 expression, when oviductal mucosa cells were co-cultured with endometrial cells at ratios of 1:1, 1:0.5, and 1:0.1, was consistent. Specifically, at the 1:1 ratio, a significant increase was observed (*P* < 0.05) (Figure 5a and b), while at the other ratios, there was an increase in expression, albeit not statistically significant (*P* > 0.05) (Figures 4 and 5). Regarding ER, no significant differences were detected across all three co-culture ratios (*P* > 0.05) (Figure 4a and b).

## 4 Discussion

Uterine adhesions pose significant physical and emotional distress to a substantial proportion of reproductive-aged women, necessitating treatment that aims to restore the uterine cavity’s normal anatomy, decrease adhesive recurrence rates, regenerate the endometrium, and ultimately enhance fertility in patients with IUA. However, current therapeutic approaches often prove insufficient or ineffective in promoting adequate endometrial regeneration following surgery in cases of severe IUA. Available evidence highlights the resemblance between tubal mucosa and endometrium in both tissue structure and functionality. Structurally, these tissues exhibit embryonic homology, being primarily composed of epithelial and stromal cells, including ciliated and secretory cells, all of which are governed by ovarian hormones and display comparable cellular morphology, albeit with varying cell ratios [17–20]. Functionally, both mucous membranes undergo hormonally-driven morphological, physiological, and biochemical alterations throughout the menstrual cycle, displaying numerous parallels that support early embryonic development and implantation [21–23]. In this study, we isolated primary endometrial and oviductal mucosa cells for *in vitro* cultivation and co-cultivated the retrieved oviductal mucosa cells with varying quantities of endometrial cells through Transwell inserts, endeavoring to mimic distinct endometrial absence scenarios within the uterine cavity. These endometrial cells, predominantly consisting of epithelial and stromal cells, were used to assess the growth of oviductal mucosa cells under these conditions. Our earlier findings indicated that oviductal mucosal cells can coexist and thrive alongside the endometrium, regardless of its quantity, without affecting their morphology or proliferation rate, and they are capable of undergoing *in vitro* meiosis.

In the present investigation, we delved deeper into the tolerance of oviductal mucosa cells following this coexistence and discovered that their tolerance was not hindered by the number of endometrial cells. Notably, when the two cell types were co-cultured in equal proportions, there was a significant increase in the expression of two crucial tolerance mediators, LIF and avβ3, which are essential for embryo implantation, with statistically significant differences (*P* < 0.05). This suggests that the tubal mucosa retains its tolerance after residing within the uterine cavity and implies that the endometrial cells might secrete certain substances that stimulate LIF and avβ3 production in the tubal mucosa cells, hence proposing that the co-culture enhances the tolerance of the tubal mucosa cells. Previous studies have shown that integrin αvβ3 is the most studied tolerogenic marker in the endometrium [24] that plays an important role in embryonic attachment [25]. Also, the literature indicates that Avβ3 is also expressed in the fallopian tube epithelium throughout the human menstrual cycle and is sharply upregulated in the mid-secretory phase, which is the endometrial tolerogenic phase, and this expression is synchronized with the endometrium [26]. These reports suggest that the tubal mucosa may have a similar or even the same tolerogenic properties as the endometrium. The expression of avβ3 was indeed detected in the tubal mucosal cells of all groups in the present study. However, unlike previous studies, the cells used in the present study for the detection of avβ3 were relatively purified oviductal mucosal stromal cells by enzymatic digestion, whereas previous studies reported expression in oviductal mucosal epithelial cells [26]. This study complements this gap, as the expression of avβ3 in oviductal mucosal stromal cells has not been reported in previous studies. The literature shows that avβ3 is also expressed in endometrial stromal cells [27]. Thus, combining previous studies with the present study further provides more evidence for the similarity between the tubal mucosa and endometrium. In addition, this study also found that the expression of avβ3 was elevated in the tubal mucosa cells when the tubal mucosa cells were co-cultured with endometrial cells in a 1:1 ratio, with a significant difference, *P* < 0.05, and there was no significant difference in the 1:0.5 and 1:0.1 ratios, and this result was consistent in RT-PCR and cellular IHC, indicating that the co-cultured tubal mucosa stromal cells had avβ3 which consistently improved from transcription to the protein synthesis stage of performing function. The mechanism of enhancement also needs further study in follow-up.

Indeed, we know that the embryo implantation process is extremely complex, with thousands of proteins, i.e., molecular languages, involved. To be able to know more about the tolerance of the tubal mucosal cells in the endometrial cell microenvironment, we performed ER, PR, and LIF tests simultaneously. Previous studies have shown that estrogen promotes endometrial proliferation, while progesterone causes secretory changes, metaplasia, and edema in the endometrium, and that these two hormones together provide a suitable environment for implantation of the embryonic follicle, acting mainly through the cognate receptors ER and PR [28–32]; LIF, on the other hand, is involved in mediating and facilitating the communication between the embryo and the endometrium through multiple pathways [33–39]. Previous studies have confirmed that PR and ER are also expressed in the fallopian tube, and that ER and PR are expressed in the nuclei of the epithelial and mesenchymal cells of the fallopian tube, with expression increasing during the proliferative phase and decreasing during the secretory phase [7].

In the present study, we found that tubal mucosal cells expressed ER and PR when cultured alone versus when cultured in different concentrations of endometrial cell microenvironment.

Observations of ER expression indicated a discernible downregulation in tubal mucosa cells when cultivated independently, as opposed to their co-culture with endometrial cells at ratios of 1:1, 1:0.5, and 1:0.1 (designated as experimental groups 1, 2, and 3). This suggests that the presence of endometrial cells may have a modulatory effect on ER expression in tubal mucosa cells. Specifically, the co-culture conditions appear to mitigate the downregulation observed in isolated tubal mucosa cells. In the context of reproductive health, estrogen plays a crucial role in regulating the menstrual cycle and maintaining endometrial function. The observed downregulation of ER in isolated tubal mucosa cells highlights the importance of cellular interactions within the reproductive tract. Endometrial cells likely provide signals that stabilize or upregulate ER expression, which could be critical for maintaining normal tubal function and fertility. This finding aligns with previous studies that have demonstrated the importance of crosstalk between different cell types in the reproductive tract, particularly in the regulation of hormone receptors.

In contrast, the expression of PR was similar whether the tubal mucosa cells were cultured alone or co-cultured with endometrial cells at ratios of 1:1, 1:0.5, and 1:0.1. There was no significant difference in PR expression between the different co-culture groups (*P* > 0.05), indicating that the presence of endometrial cells does not significantly alter PR expression in tubal mucosa cells. This suggests that while endometrial cells influence ER expression, they do not have a comparable effect on PR expression. This differential regulation underscores the complex interplay between hormonal signaling pathways in the reproductive tract. Overall, these findings provide important insights into the cellular mechanisms underlying hormonal regulation in the reproductive tract. Understanding these interactions is essential for developing targeted therapies for conditions such as infertility, uterine fibroids, and adenomyosis, where hormonal imbalances play a significant role.

Another study showed that LIF was also expressed in the human fallopian tube, while LIF in the human fallopian tube did not change much throughout the menstrual cycle, its expression was higher in the fallopian tube mucosa than in the remaining layers and was higher in the distal part of the fallopian tube, and its expression in the epithelium and mesenchyme of the fallopian tube was not regulated by ovarian hormones [40,41]. The expression in the epithelium and interstitium of the fallopian tube was not regulated by ovarian hormones. The expression and secretion of LIF were detected in all groups of cells and cell cultures in this study, which further corroborates previous studies. In addition, when compared with oviductal mucosal cells cultured alone, elevated levels were found in all groups in co-culture, with significant differences at a 1:1 ratio. In contrast, when LIF expression in oviductal mucosal cells was detected by RT-PCR, the difference was even more significant at *P* < 0.01 when the oviductal mucosal cells were co-cultured with endometrial cells in a 1:1 ratio, indicating that the more endometrial cells there were, when the two cells were co-grown, the more LIF was secreted. The mechanism of LIF elevation in tubal mucosal cells when co-cultured with endometrial cells is still unclear. Previous studies found that the expression of LIF was significantly higher in tubal pregnancy than in normal [40]. It is speculated that LIF may be involved in regulating the implantation of tubal pregnancy. Whether the mechanism of LIF elevation in the present study is similar to that of LIF elevation in tubal pregnancy needs to be further investigated.

One key limitation is the relatively small sample size of this study group. The tubal mucosa and endometrium specimens were collected from only 22 patients who underwent endometrial scraping and removal of fallopian tubes and uterus due to various conditions. Given the small sample size, the generalizability of our findings may be limited. Future studies with larger, more diverse patient populations are needed to confirm and expand upon our results.

In conclusion, our findings demonstrate that the expression of key tolerance-related markers within oviductal mucosa cells was not diminished during their co-culture with endometrial cells, and, intriguingly, the expression levels of the pivotal tolerogenic marker, avβ3, as well as LIF, were significantly upregulated at a 1:1 cell ratio. These observations imply an enhancement of the tubal mucosa’s tolerogenic capacity within the simulated uterine environment, and they tentatively propose that our approach utilizing tubal mucosa as an alternative to the endometrium for treating IUA holds promise. However, this study represents just the initial stage, and more extensive experimental evidence is required to substantiate the underlying mechanisms.

## 5 Conclusion

This study demonstrates that co-culture conditions, particularly in a 1:1 ratio, significantly enhance the expression of LIF and avβ3. While ER expression showed a non-significant downward trend, PR expression remained stable across all groups. These findings indicate that tubal mucosal cells can grow in the endometrial cell microenvironment and that co-culture improves the tolerance of tubal mucosal cells. These results suggest that the interaction between tubal mucosal cells and endometrial cells modulates critical pathways involved in IUAs. Understanding these molecular mechanisms could lead to the development of new therapeutic strategies for preventing and treating IUAs. Future research should focus on elucidating the specific roles of LIF and avβ3 in IUA prevention and exploring potential clinical applications.

## Abbreviations

ELISA: enzyme-linked immunosorbent assay
ER: estrogen receptor
IUAs: intrauterine adhesions
LIF: leukemia inhibitory factor
PCR: polymerase chain reaction
